# Cryptogenic multifocal ulcerous stenosing enteritis: a difficult diagnosis

**DOI:** 10.1111/ans.17489

**Published:** 2022-01-27

**Authors:** Chen Lew, Anshini Jain, Jonathan Chua, Alex Wong

**Affiliations:** ^1^ Medicine, Nursing and Health Sciences Monash University Melbourne Victoria Australia; ^2^ Colorectal Surgery Eastern Health Melbourne Victoria Australia

Cryptogenic multifocal ulcerous stenosing enteritis (CMUSE) is chronic and recurrent stenosis from idiopathic multifocal strictures and ulcerations of small bowel.^1^ It is a rare enteropathy with just over 60 cases in literature, often misdiagnosed as Crohn's disease or NSAID‐induced enteropathy.^2^, ^3^ The ‘gold standard’ tool for this condition is capsule endoscopy or double balloon enteroscopy.^4^ We present a 59‐year‐old man with recurrent abdominal cramps and chronic iron deficiency anaemia, diagnosed with CMUSE on histology.

**Fig. 1:**
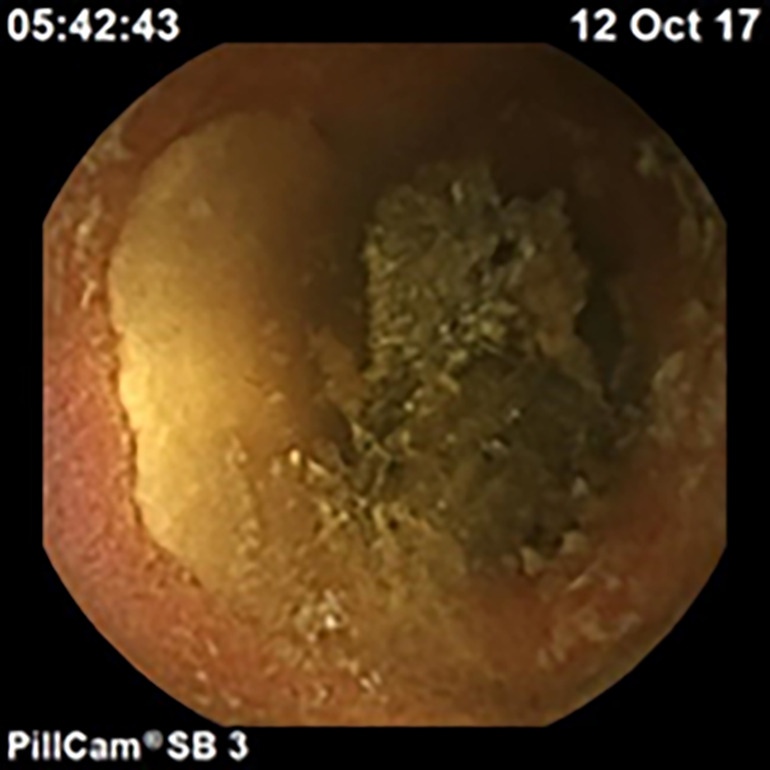
Pill endoscopy findings diagnostic of CMUSE (strictures shown).

Our patient presented acute onset bloody diarrhoea and faeculent vomiting over 1 day. He had a 10‐year history of intermittent, small‐volume bright and dark per‐rectal bleeding and iron deficiency anaemia, requiring multiple iron and blood transfusions. No pathology was evident on gastroscopy or colonoscopy, however capsule endoscopy revealed multiple circumferential ulcers with non‐obstructing stricturing distributed throughout the small bowel without active bleeding. This was attributed to NSAID‐induced enteropathy initially. He also suffered from polyarthralgia requiring long‐term NSAIDs, and symmetrical sensory neuropathy of both feet and hands with poor sensation and grip strength (Fig. 1).

Clinical examination was consistent with an acute small bowel obstruction. Oral aphthae, xerostomia, and mechanical joint pain of the ankles, hip and lower back were also observed.

Pathology revealed iron deficiency anaemia (Hb:90, MCV:70) with no raised white cells and CRP of 90 (normal:0–10). No other abnormalities.

His abdominal CT revealed ‘prominent mediastinal lymph nodes (up to 21 mm) with surrounding soft tissue stranding’. Several bilateral inguinal lymph nodes were also enlarged however no bowel dilation was seen.

**Fig. 2:**
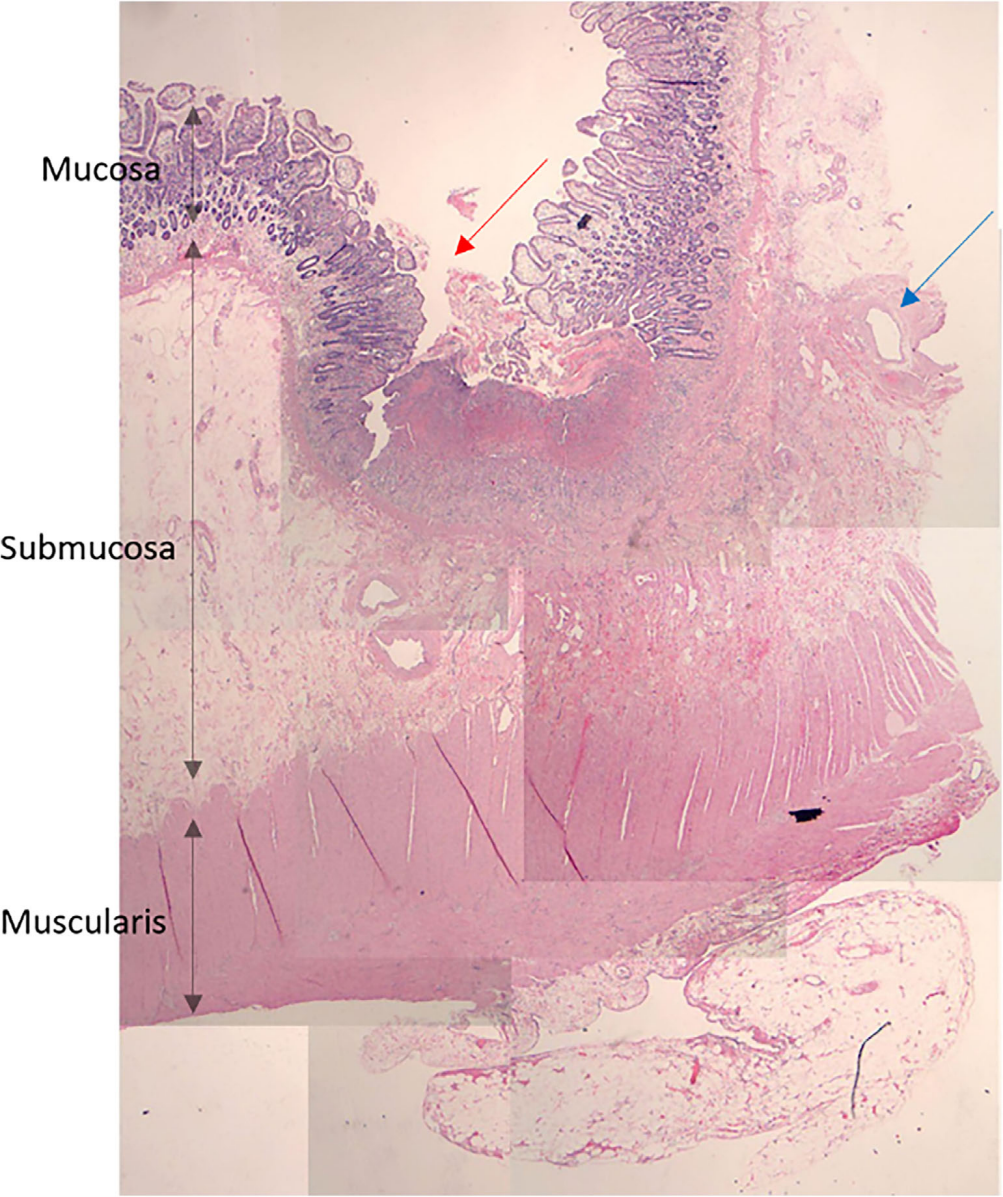
Tile image of small bowel, showing superficial erosion with vascular changes in underlying stroma. Superficial ulceration, not transmural (red) abnormal blood vessels in submucosa (blue).

He underwent an exploratory laparotomy for a provisional diagnosis of small bowel obstruction and multiple jejunal and ileal structures were discovered intraoperatively. Multiple stricturoplasties and a 5 cm ileal resection with anastomosis were performed. Histology revealed extensive segmental chronic stenosis of small bowel tissue with inflammatory strictures with arteriovenous thickening, consistent with CMUSE (Fig. 2).

Over the following months, stricturing recurred causing per‐rectal bleeding, abdominal cramping and irregular bowel actions. Five‐months later, a second laparotomy revealed over 30 strictures from mid‐to‐distal small bowel, some with significant pre‐stenotic dilation. A 80 cm of diseased small bowel was resected with hand‐sewn end‐to‐end anastomosis. Histopathological examination again confirmed CMUSE as the diagnosis.

Following this, he commenced budesonide (3 mg TDS) for 3‐months. Unfortunately, symptoms of fatigue, per‐rectal bleeding and abdominal cramping continued, and pill endoscopy revealed multiple ulcerated strictures. He had minor relief trialling exclusive enteral nutrition as he did not tolerate steroids. He has since undergone another small bowel resection performed with a cholecystectomy (due to previous ascending cholangitis) and has approximately 400 cm of small intestine remaining.

Previously, CMUSE was a diagnosis of exclusion, with steroid sensitivity and extraintestinal symptoms as the leading diagnostic factors.^5^ In our case, capsule endoscopy was utilized as the primary investigative tool, however diagnosis was confirmed with histology. Due to low incidence, clinicians may have difficulty diagnosing the condition based on endoscopy unless there is a high clinical suspicion.^3^ Table 1 demonstrates that although macroscopically similar, there are some distinguishing microscopic characteristics.

**Table 1:** Pathological findings of CMUSE and other differential diagnoses

	CMUSE	NSAID‐Induced Enteropathy	Small bowel Crohn's Disease
Macroscopic appearance	Multifocal ulcers and strictures restricted to the small bowel (colonic sparing),^6^ resulting in chronic partial obstructive symptoms.	Multiple stricture sites with semi‐circular ulcers (usually with active haemorrhage) or annular constrictions of the mucosa and submucosa leading to an obstructed lumen.^7^	Skip lesions and cobblestone appearance, often in an ileocolic distribution.^8^
Microscopic appearance	Superficial ulceration, fibrosis and non‐specific inflammation of the mucosa and submucosa, vascular changes without transmural inflammation.^6^	Ulceration (including diffuse loss of villi, mucosal and submucosal neutrophilic exudates) and transmural inflammation.^7^	Transmural inflammation with widening of the submucosa by oedema and inflammatory infiltrate, scattered aggregations of granulomatous lymphoid tissue.^8^

Characteristic symptoms of CMUSE include abdominal cramping, gastrointestinal bleeding and anaemia, with a relapsing‐remitting course despite surgery and anti‐inflammatory treatment.^1^ Extraintestinal manifestations include peripheral neuropathy, buccal aphthae, sicca syndrome, polyarthralgia, Raynauds and arterial hypertension.^9^ Our patient had long‐standing peripheral neuropathy, oral aphthae, sicca syndrome and polyarthralgia. The incidence of gallstone‐related pathology and CMUSE is currently unknown. Chung's 2015 study identified a diagnostic delay of 50.5 months after symptom onset, and given the chronic nature of this condition, any clinical clues that aid earlier diagnosis are valuable.n.^1^

CMUSE is steroid‐sensitive in majority of cases, however up to 50–66% of patients become steroid dependent.^4^, ^5^, ^10^ Koutova (2010) suggests 20 mg of prednisolone daily should reduce steroid dependency while causing regression of disease.^4^ While higher dose regimens of steroids are likely to cause increased steroid dependence, further studies to validate this in CMUSE are required.

CMUSE is a rare but important differential of benign small bowel strictures. Treatment is aimed at reducing steroid dependence and preserving small bowel length, with utilization of surgical resection for occluding strictures. This study encompasses several key clinicopathological features consistent with a diagnosis of CMUSE and aims to improve awareness to lead to earlier diagnosis.
